# A Janus Amyloid-like Nanofilm Inhibits Colorectal Cancer Postoperative Recurrence and Abdominal Adhesion via Synergistic Enzyme Cascade

**DOI:** 10.3390/nano15090670

**Published:** 2025-04-28

**Authors:** Man Zhang, Junhao Kou, Zhenyi Song, Ling Qiu, Chunzhao Yang, Qi Xue

**Affiliations:** 1 Department of General Surgery, Southern Medical University Hospital of Integrated Traditional Chinese and Western Medicine, Southern Medical University, Guangzhou 510315, China; 2 College of Pharmacy, Xi’an Medical University, Xi’an 710021, China

**Keywords:** Janus protein nanofilm, postoperative recurrence of colorectal cancer, abdominal adhesion, amyloid-like protein self-assembly

## Abstract

Postoperative peritoneal adhesion and high recurrence rates are critical challenges in the clinical treatment of colorectal cancer. In this study, based on amyloid-like protein self-assembly technology, a novel Janus protein film was developed. The protein film encapsulates glucose oxidase (GOx) and catalase (CAT), which is named PTL@GC. Through a one-step method involving cysteine-reduced lysozyme-induced amyloid-like self-assembly, the film was co-loaded with GOx and CAT to achieve synergistic anti-adhesion and anti-tumor recurrence effects. The Janus film features a hydrophobic side that stably adheres to the intestinal surface without exogenous chemical modification and a hydrophilic side that prevents adhesion. The loaded GOx selectively induces disulfidptosis in SLC7A11-overexpressing tumor cells, while CAT degrades H_2_O_2_ to alleviate hypoxia and inhibit oxidative stress, significantly reducing adhesion-related fibrosis. The experimental results demonstrate that PTL@GC exhibited excellent mechanical properties, high enzyme activity retention (>90%), and controllable degradability (complete metabolism within 50 days). In animal models, PTL@GC reduced postoperative adhesion area by 22.77%, decreased local tumor burden to 28.42% of the control group, and achieved an inhibition rate of 58.49%, without inducing systemic toxicity. This study presents a biologically safe and functionally synergistic approach to addressing dual complications following colorectal cancer surgery, offering potential insights for future research on multifunctional Janus materials.

## 1. Introduction

Colorectal cancer (CRC) is one of the most common and deadly malignant tumors worldwide. According to 2023 statistics, there are approximately 1.93 million new cases globally, resulting in 940,000 deaths [1]. Surgical resection combined with radiotherapy and chemotherapy is the standard treatment for early-stage CRC, but the 5 year postoperative recurrence rate remains as high as 30–40% [2]. Additionally, the incidence of postoperative complications such as peritoneal adhesions exceeds 93%, which can lead to intestinal obstruction, chronic pain, and infertility, with 30–50% of patients requiring secondary adhesion lysis surgery [3]. Nanotechnology has provided new ideas for multi-target collaborative treatment. A multifunctional drug delivery system based on mesoporous silica nanoparticles (MSNs) can achieve tumor microenvironment–responsive drug release by precisely delivering chemotherapeutic drugs, targeting molecules, and immunomodulators. It can effectively inhibit drug resistance pathways and reduce systemic toxicity [4]. Although there has been considerable research on independent treatment strategies for tumor recurrence or adhesions, multifunctional materials that can simultaneously address both issues are rarely reported. Therefore, developing an innovative intraoperative implantable interface material with anti-tumor recurrence, anti-adhesion, and biocompatibility functions is a critical challenge for improving the prognosis of CRC patients [5].

Currently, common postoperative anti-recurrence strategies include adjuvant chemotherapy, radiotherapy, immune checkpoint inhibitor therapy, and physical isolation measures such as local tissue fillers and biological barrier films. However, these approaches often face challenges related to systemic toxicity and local microenvironment regulation. In this context, anti-adhesion materials are an important means of synergistically preventing postoperative adhesions and tumor recurrence. Traditional postoperative anti-adhesion materials (polyethylene glycol hydrogels, chitosan films) can temporarily cover wounds but generally suffer from insufficient mechanical properties, low drug release efficiency, and short in vivo retention times [5]. For example, electrospun nanofiber films are difficult to use for tumor-targeted therapy due to poor mechanical stability and drug burst release issues, while polylactic acid (PLA) materials are limited in clinical applications due to uncontrollable degradation rates and limited drug-loading capacity [6,7]. On the other hand, tumor treatment materials often exhibit toxicity to normal cells due to a lack of tissue specificity or limited efficacy due to insufficient adaptation to the tumor microenvironment (hypoxia, oxidative stress). Notably, there is a potential link between postoperative adhesions and tumor recurrence in pathological mechanisms: accumulation of reactive oxygen species (ROS) in the inflammatory microenvironment can promote fibrosis formation and accelerate tumor cell proliferation. This suggests that regulating local redox balance may be a strategy for synergistically intervening in both complications. Currently, how to achieve adhesion inhibition and anti-tumor synergistic effects through material-mediated ROS clearance while balancing mechanical adaptability, drug release precision, and biosafety remains a core scientific problem in this field.

In recent years, Janus materials have shown significant advantages in anti-adhesion and anti-tumor fields due to their asymmetric structures and multifunctionality, effectively reducing adhesions and improving the precision and efficacy of tumor treatment [8]. Traditional Janus materials rely on complex chemical modifications such as surface grafting and plasma treatment to achieve functional partitioning, but exogenous reagents may introduce biological toxicity, and poor interface stability can lead to functional degradation. Our group’s previously published article comprehensively verified the Janus structure of the lysozyme–cysteine protein film through multidimensional experiments. First, surface chemical composition analysis (TOF-SIMS and XPS) showed that the air-contact side (CA-side) is rich in hydrophobic residues, such as phenylalanine, tyrosine, and valine, while the water-contact side (CW-side) is rich in hydrophilic residues (arginine, serine), with significantly more oxygen/nitrogen-containing groups on the CW-side than on the CA-side. Second, surface physical morphology characterization (AFM and SEM) revealed that the CA-side exhibits a flat, wrinkled morphology, while the CW-side is composed of sheet-like oligomers forming a rough surface, with the film thickness increasing over assembly time and a clear layered structure. Hydrophilicity/hydrophobicity tests (WCA) further confirmed that, after thorough drying, the contact angle of the CA-side is 120° (hydrophobic), while that of the CW-side is 40° (hydrophilic), indicating distinctly different hydrophilic/hydrophobic properties on the two sides. Molecular structure analysis (SERS and FTIR) showed strong hydrophobic group signals on the CA-side and hydrophilic group signals on the CW-side, with significant differences in secondary structures between the two sides. Mechanical property tests (nanoindentation and shear adhesion) demonstrated that the film’s Young’s modulus is as high as 8.3 ± 0.6 GPa, and the adhesion strength of the CA-side is twice that of the CW-side, with the hydrophobic side enhancing adhesion through increased interfacial contact. Finally, dynamic assembly process tracking (QCM-D and in situ AFM) revealed the layer-by-layer stacking process of sheet-like oligomers, supporting the formation mechanism of the two-sided differences. These experimental results, from chemical composition, morphology, hydrophilicity/hydrophobicity, molecular structure, mechanical properties, and dynamic assembly processes, mutually corroborate and collectively prove the Janus structure of the protein film. The doped GOx and CAT do not participate in the formation of the amyloid-like protein film, thus having no impact on the Janus structure of the film itself [9]. Our research group’s amyloid-like protein film has been applied in areas such as antibacterial urinary catheters [10], bone defect repair [11], and wound healing [12]. Preliminary studies have found that it can form a stable Janus structure through air–water interface self-assembly without any chemical modification. Moreover, two-dimensional film materials can completely cover and adhere to wound sites, making them suitable for anti-adhesion and anti-tumor recurrence applications.

**Scheme 1:**
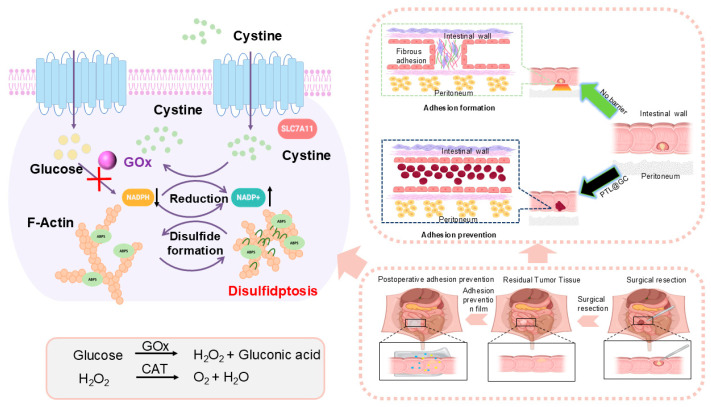
Schematic diagram of local PTL@GC application during colorectal cancer resection surgery. The illustration demonstrates two hypothesized mechanisms of action: (1) potential suppression of tumor recurrence through enzyme cascade–activated disulfidptosis, and (2) possible prevention of postoperative adhesions via the Janus characteristics of PTL@GC material.

Based on this, we designed a Janus heterogeneous self-assembly synergistic enzyme cascade strategy [13]. Using a self-assembly system of lysozyme and cysteine, we co-loaded glucose oxidase (GOx) and catalase (CAT) in a one-step process, aiming to simultaneously achieve dual functions of anti-tumor recurrence and anti-postoperative adhesion [14]. GOx can induce disulfidptosis in tumor cells and tissues with high SLC7A11 expression, effectively inhibiting tumor recurrence [15,16]. Meanwhile, CAT would catalyze the hydrogen peroxide (H_2_O_2_) produced by GOx and the H_2_O_2_ accumulated during wound healing into oxygen, alleviating the hypoxic state of the tumor microenvironment and reducing oxidative stress to mitigate postoperative inflammatory reactions, thereby further lowering the risk of adhesion formation [17,18,19]. This synergistic mechanism will enable the protein film loaded with glucose oxidase (GOx) and catalase (CAT) (PTL@GC) to exhibit significant advantages in inhibiting tumor recurrence and preventing adhesion. By combining anti-adhesion and anti-tumor recurrence properties in a biodegradable platform, PTL@GC could offer a potential strategy for improving postoperative outcomes in colorectal cancer. If validated, this approach may contribute to advancing multifunctional solutions in surgical oncology (Scheme 1).

## 2. Materials and Methods

### 2.1. Chemical and Materials

Lysozyme, GOx, and CAT were purchased from Yuanye Biotechnology Co., Ltd. (Shanghai, China). The GOx activity assay kit, CAT activity assay kit, and DCFHDA reactive oxygen species probe kit were purchased from Solarbio (Beijing, China). L-cysteine, tris (2-carboxyethyl) phosphine, and IR783 were purchased from Aladdin Biochemical Technology Co., Ltd. (Shanghai, China). The NADP^+^/NADPH assay kit was purchased from Beyotime Biotechnology (Shanghai, China). The CCK-8 assay kit, live/dead cell staining kit, and interleukin-6 (IL-6) detection kit were purchased from Servicebio Biotechnology Co., Ltd. (Wuhan, China). All other chemical reagents were purchased from Sinopharm Group (Guangzhou, China). The SW48luc and CT26.WT cell lines were obtained from ATCC (Manassas, VA, USA). BALB/c mice and BALB/c-nu/nu mice were purchased from Beijing HFK Bioscience Co., Ltd. (Beijing, China). NOD SCID mice were purchased from SPF (Beijing Biotechnology Co., Ltd., Beijing, China). Unless otherwise stated, all chemicals and reagents were of analytical grade and used according to the supplied standards. The glass substrates were all treated with Piranha solution (H_2_SO_4_:H_2_O_2_ = 7:3, *v*/*v*) at 100 °C for 6 h. Then, they were ultrasonically cleaned with ultrapure water and ethanol and dried with high-purity N_2_. During the experiment, ultrapure water was used throughout, which was provided by Milli-Q Advantage A10 (Millipore, Burlington, MA, USA).

Preparation of PTL@GC. Preparation of Solution 1: cysteine buffer solution (20 mg mL^−1^, pH = 10); Solution 2: lysozyme buffer solution (20 mg mL^−1^). First, 20 mg of Gox and 20 mg of CAT were added to 5 mL of lysozyme buffer solution (20 mg mL^−1^). Subsequently, 5 mL of cysteine buffer solution (20 mg mL^−1^, pH = 10) was added to the mixture in a 1:1 volumetric ratio. Then, 600 μL of the resulting solution was dropped onto each 24 mm × 24 mm glass slide, followed by incubation at 37 °C for 10 h. Finally, the glass slides were placed in ultrapure water, and the phase transition lysozyme nanofilm formed at the air–liquid interface was collected for further use. The buffer solutions mentioned in the manuscript are all 10 × PBS off-the-shelf solutions purchased from Solarbio. They were diluted 10-fold for use. According to the official website, their components are: NaCl: 1.36 M, KCl: 26 mM, Na_2_HPO_4_: 80 mM, KH_2_PO_4_: 20 mM.

### 2.2. Characterization and Method

Scanning Electron Microscopy Measurements. Field-emission SEM (FE-SEM) observations were conducted on a SU8020 (Hitachi High-Technologies Corporation, Tokyo, Japan). The PTL and PTL@GC samples were placed on electrically conductive adhesives and sprayed with gold. Cross-sectional samples of PTL@GC were subjected to wetting-off in liquid nitrogen [20,21]. ELMI (Conductive Adhesive): Nissin Double-sided Conductive Adhesive FN731-5N (5 mm wide).

X-ray Photoelectron Spectroscopy Measurements. X-ray photoelectron spectroscopy (XPS) of the lysozyme and PTL@GC samples was performed using an AXIS ULTRA (Kratos Analytical Ltd., Manchester, UK) [22].

Water Contact Angle Measurement. The water contact angle (WCA) of PTL and PTL@GC was measured on an OCA 20 (Data Physics Instruments GmbH, Filderstadt, Germany) [23].

Fourier Transformed Infrared Spectroscopy Measurements. Fourier transformed infrared (FTIR) spectra were obtained using a Vertex 70 V spectrometer (Bruker Corporation, Billerica, MA, USA). FTIR spectra were obtained between 400 and 4000 cm^−1^ with a resolution of 1 cm^−1^, using the KBr disk method [24].

Laser Scanning Confocal Microscopy Measurements. Thioflavin T (ThT)-stained PTL@GC was tested using an Olympus laser scanning confocal microscope FV 1200 apparatus (Olympus Corporation, Tokyo, Japan). The activator was 405 nm [25]. Numerical Aperture: 5×/0.15 NA.

Pinhole Aperture: 1 AU. Filters: excitation wavelength: 405 nm; emission wavelength: 425–475 nm (bandpass).

Circular Dichroism Spectrum Measurement. Far-UV circular dichroism (CD) (JASCO Corporation, Tokyo, Japan) spectra were collected under a constant nitrogen flush at 25 °C and recorded at 2.0 nm from 200 to 260 nm [26].

Monitoring the Changes in Protein Hydrophobic Regions Using an ANS Fluorescence Probe. In a 96-well plate, 20 μL of an ANS (8-anilino-1-naphthalenesulfonic acid, 200 μM) solution was mixed with 90 μL of different protein solutions, including lysozyme (20 mg mL^−1^), GOx (2 mg mL^−1^), and CAT (2 mg mL^−1^). The mixed solutions were then placed in a microplate reader, and 90 μL of a cysteine (20 mg mL^−1^) solution was added. The fluorescence intensity of the samples was recorded over time using an excitation wavelength of 355 nm and an emission wavelength of 470 nm. This method utilizes the selective binding of the ANS fluorescent probe to hydrophobic protein regions, allowing for the monitoring of conformational changes of the proteins upon the addition of cysteine. Time-dependent changes in the fluorescence signal can provide insights into the structural dynamics of the proteins [27].

Tryptophan (Trp) Fluorescence Monitoring. First, 90 μL of a cysteine solution (20 mg mL^−1^, pH = 10) was separately mixed with 90 μL of different protein solutions, including lysozyme (20 mg mL^−1^), GOx (2 mg mL^−1^), and CAT (2 mg mL^−1^). Then, the fluorescence intensity of the samples was recorded over time using a Spark^®^ multimode microplate (Tecan Group Ltd., Männedorf, Switzerland) reader until the fluorescence intensity remained unchanged. The excitation wavelength was set at 285 nm, and the emission wavelength was set at 340 nm.

Monitoring Changes in Protein Sulfhydryl Groups Using NPM Fluorescence Labeling. In a 96-well plate, 20 μL of an N-(1-pyrenyl) maleimide (NPM, 10 mmol L^−1^ in DMF) solution was separately mixed with 90 μL of different protein solutions, including lysozyme (20 mg mL^−1^), GOx (2 mg mL^−1^), and CAT (2 mg mL^−1^). Then, 90 μL of a cysteine solution (20 mg mL^−1^, pH = 10) was added to the mixtures. The fluorescence intensity of the samples was recorded over time using a microplate reader. The excitation wavelength was set at 330 nm, and the emission wavelength was set at 380 nm [28].

MALDI-TOF-MS Characterization of Covalent Conjugation (Bruker Daltonics GmbH, Bremen, Germany). A lysozyme solution (20 mg mL^−1^) was mixed in equal volume with an IR783 solution (2 mg mL^−1^), and the resulting reaction solution was diluted 10-fold. Next, 1 μL of the diluted mixture was combined with an equal volume of an α-cyano-4-hydroxycinnamic acid solution (10 mg mL^−1^ in tetrahydrofuran) and thoroughly mixed. The mixture was then spotted onto the MALDI target plate, dried, and subjected to matrix-assisted laser desorption/ionization time-of-flight (MALDI-TOF) mass spectrometric analysis to detect changes in the molecular mass of lysozyme before and after the conjugation reaction.

The PTL@GC protein film was placed inside an in vivo imaging system (Smart-LF, Seoul, Republic of Korea), and the fluorescence of the material was imaged using the ICG imaging mode. This method utilizes an in vivo imaging system to detect the fluorescence signal emitted by the PTL@GC protein film.

Determination of Drug Encapsulation Efficiency of Sustained-Release Coating. To ensure complete dissolution of the drug, the prepared PTL@GC was immersed in a 1% ascorbic acid solution and stirred vigorously at room temperature (700 rpm) for 4 h. The resulting solution was then centrifuged (10,000 rpm, 10 min), and the supernatant was collected. The absorbance of the supernatant was measured using a UV spectrophotometer (Shimadzu Corporation, Kyoto, Japan) to determine the encapsulated content of GOx and CAT in the drug delivery system.

The encapsulation efficiency (EE) was calculated using the following formula:

$$EE=\frac{W}{W_{0}}\times 100\%$$

where *W*_0_ represents the initial amount of drug loaded and *W* is the actual amount of drug stored in the sustained-release coating.

Drug Release. Using FITC-grafted CAT and GOx, we established a standard curve. The PTL@GC was soaked in 100 mL of PBS at 37 °C. Liquid samples were collected at fixed time points. We collected 0.5 mL of PBS at 0 h, 12 h, 24 h, 48 h, 72 h, 96 h, and up to 700 h, and then replenished the total volume to 2 mL. After collecting the samples, we measured the absorbance at a wavelength of 450 nm using a UV-Vis spectrophotometer to calculate the amount of drug released. 

### 2.3. Testing of Mechanical Properties

Friction Stability Test. The PTL@GC was fixed to a slider with a mass of 200 g, which was then inverted and placed on sandpaper of different roughness (grit numbers 240 or 400). Under the application of external force, the slider was moved forward by 6 cm each time. After a varying number of friction movements, the water contact angle on the surface was measured.

Bending Stability Test. The PTL@GC was prepared on a PET sheet, which was then cut into rectangular pieces measuring 1 × 5 cm^2^. The pieces were subjected to a 180° bend using a tensile testing machine. After a varying number of bending cycles, the contact angle on the surface was measured to assess the bending stability of the superhydrophobic surface.

Nano-Scratch Test. The nano-scratch test was conducted to evaluate the mechanical properties of the PTL@GC using a nano-scratch tester (NTS3, Anton Paar, Anton Paar GmbH, Graz, Austria). The scratch test was performed by applying a progressively increasing load, starting from zero, at a constant rate. During the test, the tangential force (Fx) and normal force (Fz) were continuously recorded. Each test was repeated three times to ensure reproducibility, and average values are reported [23].

$$\mu =\frac{Fx}{Fz}$$

Adhesive and Stability Testing of PTL@GC. The PTL@GC was attached to the colon of BALB/c mice. An 8 cm segment of the colon containing the material was excised and placed in 4 °C PBS buffer. Samples were collected 0, 5, 10, and 15 days post-implantation. At each time point, the colon tissue was placed in an in vivo imaging system, and the fluorescence intensity was quantitatively measured. BALB/c mice, n = 3.

### 2.4. Cell Experiments

Cell Culture. Subsequent experiments involved in vitro culture of human colorectal adenocarcinoma cells (SW48luc) and murine CRC cells (CT26). WT.SW48luc cells were purchased from WanWu Biotechnology Co. (Hefei, China) and CT26.WT-luc-gfp-puro cells were purchased from Warner Bio (Wuhan, China) Co., Ltd. The SW48luc and CT26.WT cells were incubated in high-glucose liquid culture medium (DMEM, Cytiva, Marlborough, MA, USA) with 10% fetal bovine serum (FBS, Evergreen, Shanghai, China) and 1% (*v*/*v*) antimicrobial of penicillin/streptomycin (MACKLIN) at 37 °C in a humidified atmosphere with 5% CO_2_. During cell culture, the PTL and PTL@GC used were generated between cells, and the solutions needed were filtered through a sterile filter.

Cell death and viability assays. The effects of different materials on CT26.WT, SW48luc, HUVEC, and IMR90 cells were analyzed using a CCK-8 assay kit (Servicebio). The four cell lines mentioned above were seeded into a 96-well plate at a density of 1 × 10^4^ cells/well and co-cultured for 24 h. Subsequently, the optical density (OD) of the culture medium was measured at 450 nm using an automatic microplate reader (Tecan Group Ltd., Männedorf, Switzerland). Cell viability was determined as follows:

$$Cellviability\left(\%\right)=(\frac{Abs_{experimental}-Abs_{blank}}{Abs_{control}-Abs_{blank}}\times 100\%)$$

Measurement of Reactive Oxygen Species (ROS): First, 6 × 10^4^ cells per well were inoculated intp a 48-well plate and cultured until the logarithmic growth phase. Then, except for the control group, 300 μL of the treatment solution (500 μL of 3% H_2_O_2_ + 20 mL + 1000 μL of H_2_O_2_ stock solution) was added to each well and incubated for 8 h. Subsequently, the treatment solution was removed, and the cells were washed three times with PBS to completely remove the residual liquid. Immediately afterwards, the cells were co-incubated with different materials (no materials were added to the control group or the H_2_O_2_ group) overnight. 2′,7′-dichlorodihydrofluorescein diacetate (DCFH-DA) was dissolved in serum-free medium and diluted at a ratio of 1:1000 to prepare the working solution. The cell culture medium was aspirated, the cells were washed three times with phosphate-buffered saline (PBS), 500 μL of working solution was added to each well, and the cells were incubated at 37 °C with 5% carbon dioxide (CO_2_). After 30 min of incubation, the cells were washed three times again with PBS to thoroughly remove the residual probe. ImageJ 1.53t software (Bethesda, MD, USA) was used to conduct a quantitative analysis of the images captured by an inverted fluorescence microscope. To eliminate the error caused by the increase in the number of cells, the average fluorescence value was divided by the total number of cells, and normalization processing was performed.

Live/Dead Cell Staining Experiment. A 48-well plate was seeded with 6 × 10^4^ cells per well that were treated with different materials. Subsequently, live/dead cell staining analysis using Calcein-AM and propidium iodide (PI) was conducted to investigate the synergistic effects. Images were captured using an inverted fluorescence microscope. Quantitative analysis of the images was performed using ImageJ software.

NADP^+^ and NADPH measurements. Using an NADP^+^/NADPH Assay Kit (Beyotime), total NADP^+^/NADPH levels were first measured according to the instructions, followed by separate quantification of NADPH. Utilizing the obtained total amounts of NADP^+^ and NADPH from the first two steps, the quantity of NADP^+^ in the sample, as well as the ratio of NADP^+^/NADPH, was determined [29].

Hemolysis assay. The experiment included a negative control (physiological saline), two positive controls (distilled water and 0.1% Triton X-100), PTL, and PTL@GC, with each group tested in triplicate. After co-incubation with 2% sheep red blood cells for 4 h, the supernatant was collected by centrifugation (Thermo Scientific Sorvall Legend Micro 21, Thermo Fisher Scientific, Waltham, MA, USA) (10,000 rpm, 2 min) and added to a 96-well plate. The absorbance at 542 nm was measured using a microplate reader.

SW48-luc cells were seeded at a density of 1000 cells per well in a six-well plate. Subsequently, 1 mL of either PTL or PTL@GC 24-h extract was added, along with 2 mL of complete culture medium. The cells were cultured for 20 days, with the culture medium replaced every 2 days. Finally, crystal violet and formaldehyde were used for staining and fixation, respectively. The cell colonies were counted and analyzed using ImageJ software.

### 2.5. Animal Experiments

Cecal Injury Model: BALB/c mice aged 6–8 weeks were allowed to adapt to the standard environment for 1 w. Sterilized surgical instruments, anesthetics, iodophor, and other drugs were prepared. After anesthetizing the mice by inhaling isoflurane, the abdominal area was disinfected, and an incision was made along the midline to expose the abdominal cavity. The cecum was pulled out, and a surgical scalpel was used to scratch the mucosal layer of the cecum, with the injury area being 0.5 cm^2^. After the operation, the cecum was rinsed and replaced, and the wound was sutured. The mice were transferred to a suitable environment after the operation and closely observed to ensure their nutritional intake.

Classification of adhesions according to Zühlke et al.:

Grade 0: No adhesions or insignificant adhesions.

Grade 1: Adhesions that are filmy and easy to separate by blunt dissection

Grade 2: Adhesions where blunt dissection is possible but some sharp dissection necessary, beginning vascularization.

Grade 3: Lysis of adhesions possible by sharp dissection only, clear vascularization.

Grade 4: Lysis of adhesions possible by sharp dissection only, organs strongly attached with severe adhesions, damage of organs hardly preventable.

Scoring process: The scoring was independently completed by two physicians who had received unified training. The observation time point was the 14th day after the operation. A double-blind design was adopted during the operation, and the scorers did not participate in the grouping operation. If the scoring difference was ≥2, a third senior researcher reviewed the scores and reached a consensus. Meanwhile, the adhesion site (intestine-peritoneum) and vascular distribution were recorded.

Establishment of Orthotopic Residual Colon Cancer Tissue Model. SW48luc cells were subcutaneously inoculated at a concentration of 3 × 10^6^ cells mL^−1^ into the right flank of BALB/c-nu/nu mouse. Once the subcutaneous tumor reached a volume of 50 mm^3^, colonic transplantation was performed. For the transplantation procedure, NOD SCID mice were anesthetized, and the abdomen was sterilized with iodine and alcohol swabs [30]. A small midline incision was made, and the colorectal part of the intestine was exteriorized. The serosa at the site where the tumor fragments were to be implanted was removed. Tumor fragments of 1 mm^3^ in size were implanted onto the intestinal wall. An 8-0 surgical suture was used to penetrate these small tumor fragments and secure them to the intestinal wall. The intestine was then returned to the abdominal cavity, and the abdominal wall was closed with 7-0 surgical sutures. The animals were kept in a sterile environment. Mice with successfully engrafted tumors underwent 90% tumor resection, leaving 10% residual tumor, followed by intestinal anastomosis. The model mice were divided into three groups, each consisting of five mice: control, PTL, PTL@GC. The control group received no additional treatment, while the PTL and PTL@GC groups had their respective PTL and PTL@GC adhered and completely covered on the residual tumor tissue. Finally, 6-0 sutures were used for the rectus abdominis muscle and 4-0 sutures for the abdominal wall, establishing models of orthotopic CRC and post-resection residual tumor [31].

In Vivo Degradation and Stability. IR783-labeled PTL and PTL@GC were adhered to the colon tissue of mice, and quantitative measurements of fluorescence intensity in vivo were conducted using an in vivo imaging system (VISQUE InVivo Smart-LF) at 0, 1, 2, 3, and 4 weeks [32]. Subsequently, the IR783-labeled PTL and PTL@GC, along with surrounding tissues, were harvested for histological examination, including H&E staining and IL-6 analysis [33]. Confocal microscopy was utilized to capture images of the tissues to observe the degradation of IR783-labeled PTL and PTL@GC.

## 3. Results

### 3.1. Preparation and Characterization of PTL@GC

**Figure 1:**
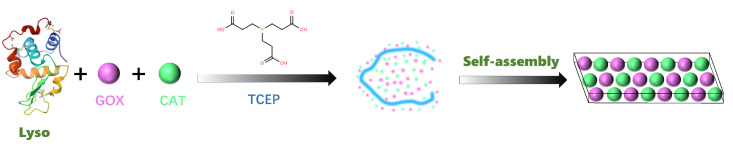
Schematic diagram of the specific synthesis process of PTL@GC.

**Figure 2:**
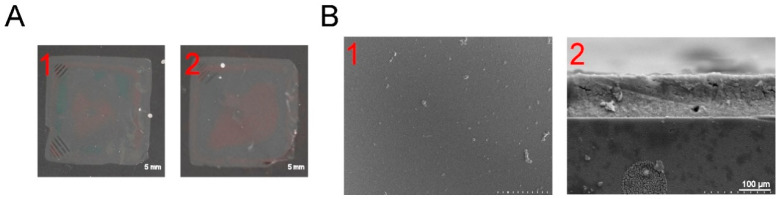
The morphologies of PTL and PTL@GC. (**A**) Optical photographs of PTL and PTL@GC. (**B**) SEM images of PTL@GC: (**B1**) surface, (**B2**) cross-section.

**Figure 3:**
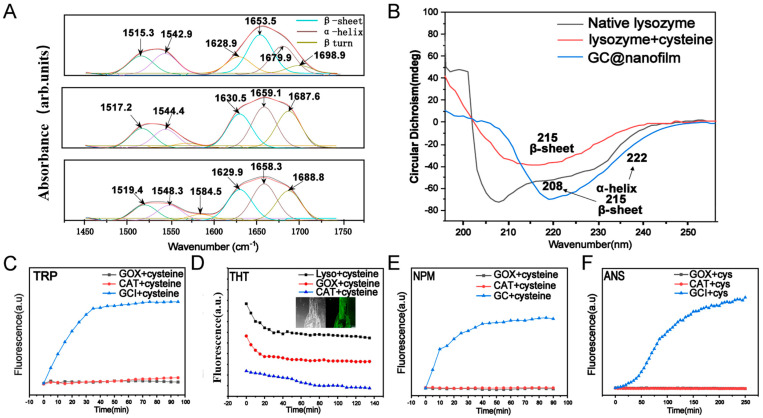
Spectral characterization during the synthesis process of PTL@GC. (**A**) Infrared spectra of lysozyme, PTL, and PTL@GC (from top to bottom). (**B**) CD spectra of lysozyme, PTL, and PTL@GC. (**C**) CRP fluorescence spectra. (**D**) ThT fluorescence spectra and confocal images. (**E**) NPM fluorescence images. (**F**) ANS fluorescence images.

When lysozyme is dissolved in water at pH 7.0 and cysteine (pH = 10, 20 mg mL^−1^) is added, the conformation of lysozyme rapidly changes (Figure 1). Cysteine disrupts its disulfide bonds, inducing the formation of amyloid-like aggregates, ultimately resulting in the formation of the PTL protein film (Figure 2(A2)). After adding GOx (2 mg mL^−1^) and CAT (2 mg mL^−1^ to the lysozyme solution (20 mg mL^−1^), cysteine selectively reacts with lysozyme without reacting with GOx or CAT, generating PTL@GC (Figure 2(A1)). Scanning electron microscopy (SEM) revealed the surface morphology of the PTL@GC film (Figure 2(B1)) and a cross-sectional thickness of approximately 620 nm (Figure 2(B2)). The secondary structure information of the amide I band was analyzed using the Peak Fit Version 4.12, (San Rafael, CA, USA) software program Peak Fit. In the peak-fitting results of the Fourier Transform Infrared (FTIR) spectrum, the enhancement of the infrared absorption peaks at 1628 and 1696 cm^−1^ indicates that it mainly has a β-sheet conformation. Compared with native lysozyme, the content of β-sheet structures in both PTL and PTL@GC increased (Figure 3A). As proven by the strong negative peak at 215 nm in the far-ultraviolet Circular Dichroism (CD) spectrum (Figure 3B), it is in a β-sheet-dominated conformation. This further confirms formation of the PTL@GC film, that is, the transformation of lysozyme from an α-helix to a β-sheet. Additionally, time-resolved fluorescence tests monitored by TRP, ThT, NPM, and ANS staining showed that lysozyme rapidly undergoes amyloid-like aggregation, reaching a growth plateau within 120 min (Figure 3C–F). Notably, GOx and CAT do not react with cysteine, ensuring high retention of their enzymatic activity. Based on our previous research, XPS elemental analysis of PTL@GC revealed that various functional groups are exposed on its surface, including C-H/C-C, C-N, C-O, C-S, O=C-N, and O=C-O (Figure S1). These groups, derived from hydrophilic and hydrophobic amino acid residues, bind to the material surface through coordination bonds, electrostatic interactions, hydrogen bonds, and hydrophobic interactions, effectively promoting the adhesion of the film to various substrates. The results indicate that the hydrophobic properties of PTL@GC are not significantly altered compared to PTL.

### 3.2. Enzyme Activity Retention and Wet Surface Adhesion Stability of PTL@GC

**Figure 4:**
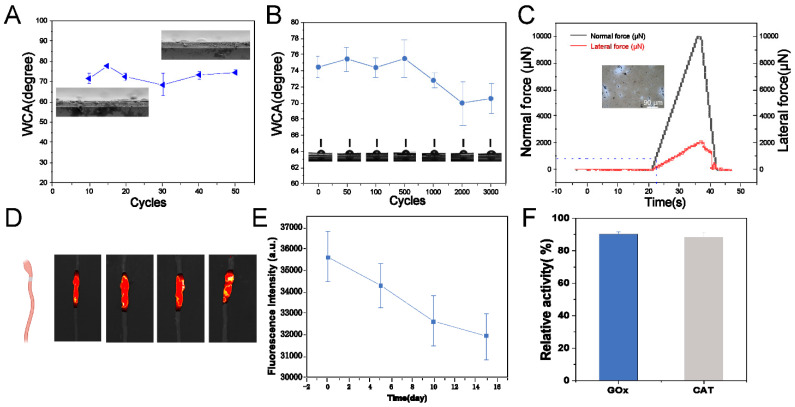
Performance of PTL@GC. (**A**) Friction stability test; (**B**) bending stability test contact angle results; (**C**) nano-scratch test; (**D**) small animal fluorescence in vivo imaging; (**E**) intestinal fluorescence quantification in vitro; (**F**) enzyme activity statistics of GOx and CAT.

The PTL@GC film exhibits superior drug-loading capacity, with CAT loading at 9.12 μg cm^−2^ and GOx loading reaching 24.03 μg cm^−2^. Enzymes encapsulated in the phase-transition protein film must retain their biocatalytic activity, which is crucial for therapeutic efficacy. We evaluated the enzymatic activity of GOx and CAT in PTL@GC using a double-antibody sandwich assay. The results showed that the enzymatic activities of both GOx and CAT remained above 90%, demonstrating excellent enzyme activity retention (Figure 4F). Considering that our treatment involves in vivo drug delivery, drug release experiments were conducted on PTL@GC. The release of GOx and CAT reached approximately 60% over 4 weeks (Figure S2), with average daily release amounts of 0.23 μg cm^−2^ for active GOx and 0.44 μg cm^−2^ for CAT. This indicates that the released GOx and CAT from PTL@GC can effectively function during the treatment period, enabling sustained drug delivery. GOx decomposes glucose in the tumor microenvironment into hydrogen peroxide, while CAT decomposes H_2_O_2_ inside and outside the tumor into oxygen, alleviating the hypoxic tumor microenvironment.

Preventing postoperative adhesions and recurrence requires PTL@GC to possess excellent mechanical stability, wear resistance, and wet surface adhesion properties. Therefore, we conducted friction stability tests, bending stability tests, nano-scratch tests, and in vitro intestinal motion simulation experiments. After 50 cycles of friction with 200-grit sandpaper, the contact angle remained within the range of 74 ± 1.8°, and no delamination or damage was observed in the cross-sectional morphology (Figure 4A). After 0 to 3000 bending cycles using a universal material testing machine, the contact angle decreased only from 74 ± 2.1° to 70 ± 1.1°, with a decay rate of only 5.4% (Figure 4B). The friction stability and bending stability test results showed that, according to medical device material industry standards, PTL@GC exhibits excellent mechanical stability and is fully capable of withstanding intestinal and abdominal wall friction and movement. The nano-scratch test is a suitable method for evaluating the adhesion and wear resistance of PTL@GC. Normal force (Fz) and friction coefficient (μ) are important indicators of adhesion and wear resistance. The experimental results showed that PTL@GC has an Fz of 0.841 mN and a friction coefficient (μ) of 0.165 (Figure 4C), demonstrating wear resistance comparable to or better than many excellent wear-resistant films or coating materials. For example, the friction coefficient of HAP coating is 0.750, and that of MWCNTs is 41.2 ± 2.1 [34,35].

To further evaluate the adhesion and stability of PTL@GC on intestinal tissue, an in vitro intestinal motion simulation was conducted. In the in vitro motion simulation experiment, BALB/c mice were euthanized, and colon tissue was extracted. PTL@GC film was prepared using lysozyme grafted with IR783 fluorescent molecules, which facilitated later observation and fluorescence quantification. The film was adhered to the isolated intestines of BALB/c mice and placed in physiological saline at 4 °C (with 1.25% penicillin added), with rotation at 120 rpm. Two additional intestinal segments were placed in each beaker to simulate mutual contact and peristalsis of the intestines. The adhesion of PTL@GC was monitored by measuring fluorescence intensity and area on days 0, 5, 10, and 15. The results (Figure 4D,E) showed a slow decrease in fluorescence signal and area on the intestines of BALB/c mice, indicating that PTL@GC exhibits excellent wet interface adhesion on the intestinal surface.

### 3.3. Selective Cytotoxicity and Disulfidptosis of PTL@GC

**Figure 5:**
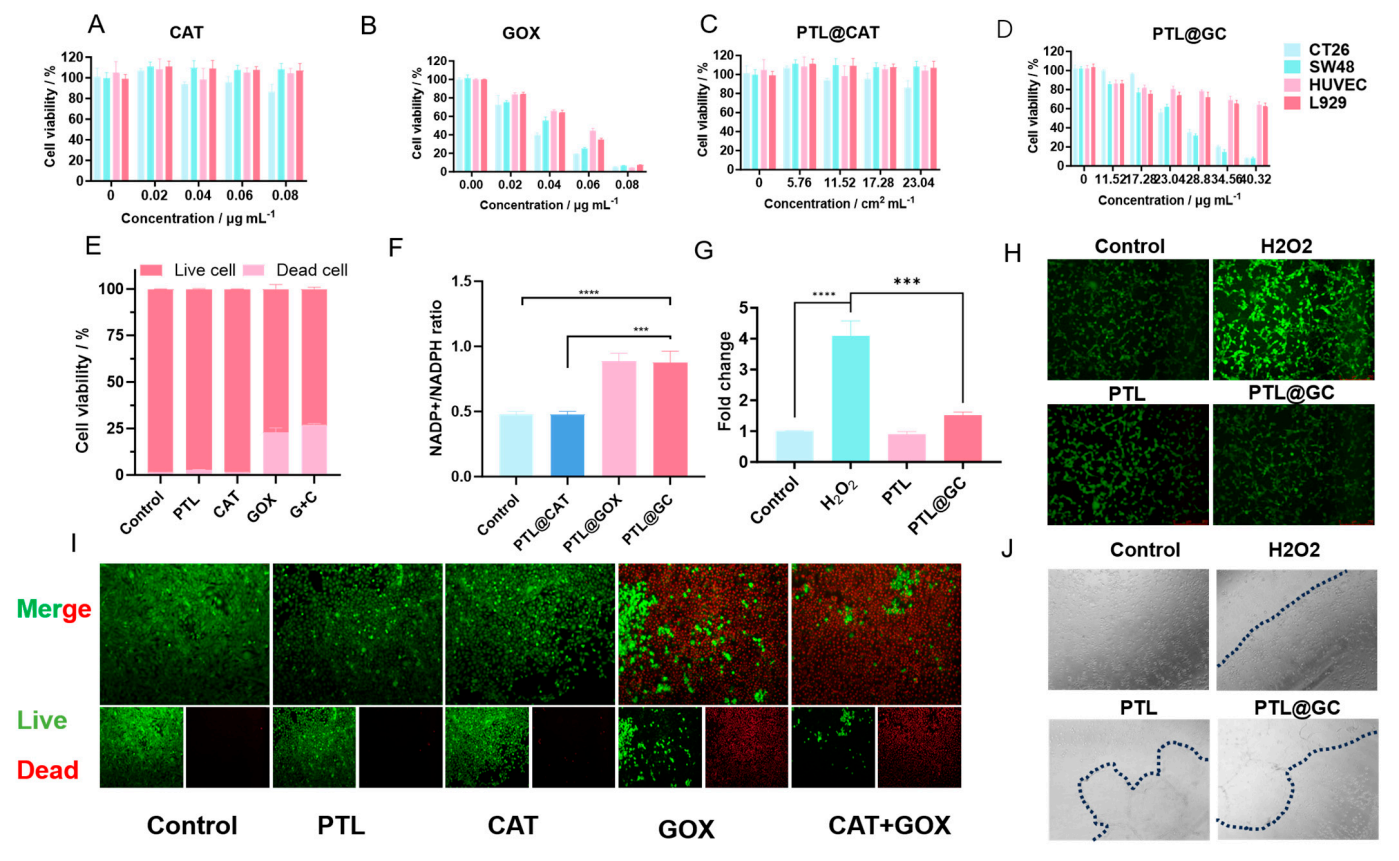
Cellular-level experiments with PTL@GC: (**A**) proliferation rate of CT26, SW48, HUVEC, and L929 cells after 12 h of treatment with different concentrations of GOx; (**B**) proliferation rate of cells after 12 h of treatment with different concentrations of CAT; (**C**) proliferation rate of cells after 12 h of treatment with different concentrations of PTL@CAT; (**D**) proliferation rate of cells after 12 h of treatment with different concentrations of PTL@GC; (**E**) live/dead cell staining statistics; (**F**) NADP⁺/NADPH ratio under different treatment conditions; (**G**) ROS fluorescence intensity statistics; (**H**) inverted fluorescence microscopy images of HUVEC cells; (**I**) live/dead cell staining and inverted fluorescence microscopy images of SW48 cells under different treatment conditions; (**J**) cell adhesion of HUVEC cells under different treatment conditions. (*** *p* < 0.001, **** *p* < 0.0001).

We evaluated the cytotoxicity of PTL (concentration range: 5.76 cm^2^ mL^−1^ to 23.04 cm^2^ mL^−1^) on tumor cells and normal cells (Figure S3). The results showed that PTL had minimal impact on normal cell growth. When co-cultured with free CAT (concentration: 0.02 to 0.08 µg mL^−1^) for 4 h, cells exhibited normal growth (Figure 5A). However, when co-cultured with free GOx (concentration: 0.02 to 0.08 µg mL^−1^) for 4 h at a concentration of 0.06 µg mL^−1^, over 81% of the cells died, while at 0.04 µg mL^−1^, tumor cell mortality was significantly higher than that of normal cells (Figure 5B). Similar results and trends were observed with PTL@GOx [36]. This phenomenon is attributed to the Warburg effect, where tumor cells rely more on anaerobic glycolysis during metabolism, leading to increased glucose consumption. PTL@CAT showed similar results to free CAT, with no effect on normal cell growth (Figure 5C).

Next, we tested the inhibitory effect of PTL@GC on cells. Based on drug loading and release rate calculations, after 24 h of co-incubation with cells, the cumulative release of GOx in different gradient treatment groups (depending on the total amount of GOx in the film) was approximately 0.02 µg, 0.03 µg, 0.04 µg, 0.05 µg, 0.06 µg, and 0.07 µg. Compared to free GOx at the same concentration, the inhibitory effect of PTL@GOx on colon cancer cell lines was only slightly reduced, maintaining the basic therapeutic effect. This phenomenon may be due to the encapsulation of GOx in PTL, which reduces catalytic sites and is related to sustained release. The continuous release characteristics of the film allow for long-term, slow release of the enzyme, improving drug utilization and reducing potential peritoneal irritation caused by burst release. Cloning experiments also showed that, compared to the control group, the inhibition rate of cells in the PTL@GC group was 75.51% (Figure S4). Live/dead cell staining results indicated that the mortality rates of SW48 cells treated with PTL@G and PTL@GC were similar (22.88 vs. 26.91), with no statistical difference (Figure 5E,I).

Additionally, GOx can induce disulfidptosis in cell lines with high SLC7A11 expression. After co-culturing PTL, PTL@G, PTL@C, and PTL@GC for 4 h, the results showed that the NADP⁺/NADPH ratio in the PTL@G and PTL@GC groups significantly increased, indicating that GOx significantly reduced NADPH levels (Figure 5F). Furthermore, when SW48 cells were co-cultured with PTL@GC, cell shrinkage was observed. Upon adding DTT, 2ME, and TCEP, the cell morphology returned to normal (Figure S5) [37]. This confirms that PTL@GC indeed induced disulfidptosis in SW48 cells.

CAT not only alleviates the hypoxic tumor microenvironment but also consumes the produced H_2_O_2_. Experimental results also confirmed this hypothesis (Figure 5G,H). H_2_O_2_ production in HUVEC cells treated with PTL@GC decreased by 34.36%, showing a significant difference compared to the positive control group. We also observed that, when HUVEC cells were added to culture dishes pre-coated with PTL and PTL@GC, cells in the PTL group adhered to the film, but the adhesion time was longer compared to areas without the film. The number of cells adhering to the PTL@GC film was significantly reduced, possibly because GOx affected the adhesion and growth of HUVEC cells to some extent (Figure 5J).

### 3.4. Biocompatibility and Degradation of PTL@GC Film

**Figure 6:**
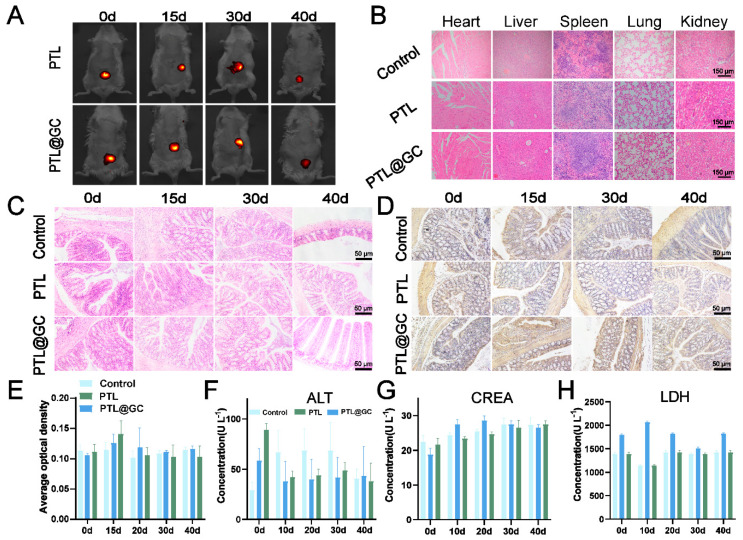
Degradation stability and biosafety evaluation of PTL@GC in mice. (**A**) In vivo imaging of IR783-labeled PTL and PTL@GC on days 10, 15, 30, and 40. (**B**) H&E staining of heart, liver, spleen, lung, and kidney tissues from the control, PTL, and PTL@GC groups. (**C**) H&E staining of intestinal tissues at different time points in each treatment group. (**D**) IL-6 immunohistochemical staining of intestinal tissues at different time points in each treatment group. (**E**) Quantitative analysis of IL-6 immunohistochemical fluorescence in intestinal tissues. (**F**) ALT levels in each group. (**G**) CREA levels in each group. (**H**) LDH levels in each group.

The biosafety and appropriate degradation time of implants are crucial for therapeutic efficacy. Therefore, this study first conducted a hemolysis assay to preliminarily evaluate whether PTL@GC is suitable for in vivo treatment. The results showed that PTL@GC exhibited almost no hemolysis even at a high material concentration of 34.56 cm^2^ mL^−1^, and PTL demonstrated similar results (Figure S6). Additionally, PTL@GC could stably adhere to the surfaces of isolated heart, liver, spleen, lung, and kidney tissues (Figure S7). In subsequent in vivo experiments, to dynamically observe the degradation process of the protein film in a living state, IR783-labeled PTL@GC and PTL were adhered to the intestinal surface of anesthetized BALB/c mice. The results indicated that both PTL@GC and PTL remained stable at the colon site for over 30 days (Figure 6A). Fluorescence significantly decreased by day 40 and almost disappeared by day 50 (Figure S8). Quantitative analysis of fluorescence intensity revealed significant degradation on days 10 and 40, with no significant changes at other time points. These findings demonstrate that the PTL@GC protein film can stably exist, perfectly meeting the requirements for postoperative colon cancer treatment and anti-tissue adhesion. H&E staining results showed normal morphology and structure in the heart, liver, spleen, lung, and kidney of the mice (Figure 6B). The body weight of mice in all groups steadily increased without any decline (Figure S9). The intestinal tissue structure and cell morphology were normal, as shown by H&E staining (Figure 6C), and IL-6 immunohistochemical staining further confirmed its biosafety. Compared to the control group, PTL and PTL@GC did not induce inflammation in the intestinal tissue (Figure 6D), and the area of this region gradually decreased over time (Figure S10), which is related to the macrophage response. Quantitative analysis of IL-6 levels showed that the AOD values of the PTL and PTL@GC groups were within the normal range, with no significant difference compared to the control group. These results collectively prove that PTL and PTL@GC protein films exhibit excellent biocompatibility and an appropriate degradation cycle. Blood biochemical indicators of mice on days 0, 10, 20, 30, and 40 were all within the normal range (Figure 6E–H and Figure S11). Subcutaneous implantation of PTL@GC also demonstrated excellent biocompatibility and degradation stability (Figures S12 and S13).

### 3.5. Anti-Adhesion Capability of PTL@GC Film

**Figure 7:**
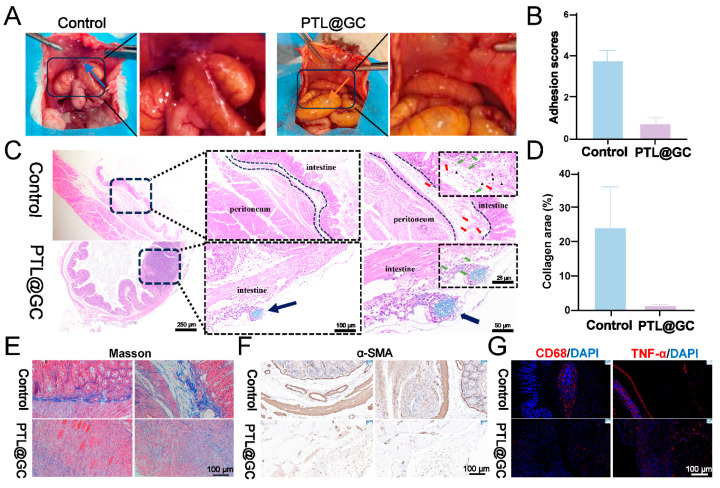
Therapeutic efficacy of PTL@GC in preventing postoperative adhesion in a mouse cecal abrasion model. (**A**) Representative images of intestinal adhesion in mice treated with PTL@GC. Untreated animals served as the control group. Blue arrows indicate adhesion sites; orange arrows indicate PTL@GC. (**B**) Adhesion scores based on visual observation. (**C**) H&E staining of intestinal adhesion tissues. Black dashed lines indicate adhesion between the cecum and abdominal wall; red arrows: blood vessels; green arrows: inflammatory cells; black triangles: collagen; black arrows: residual IR783-labeled PTL@GC. (**D**) Statistical analysis of adhesion area in different groups. (**E**) Masson’s trichrome staining of tissue sections from different groups. (**F**) Immunohistochemical staining of α-SMA. (**G**) Immunofluorescence staining of CD68^+^ (red), TNF-α (red), and DAPI (blue) in adhesion tissues (control group) and cecal tissues (PTL@GC-treated group).

The anti-adhesion efficacy of PTL@GC was first evaluated using a mouse cecal abrasion model (Figure S14). On day 0, the abdominal cavity was opened, and the mucosa was scraped with a blade until bleeding points appeared. After applying PTL@GC, the incision was sutured with absorbable sutures. Mice with untreated cecal abrasions served as the control group. On day 7 post-surgery, the abdominal cavity was reopened to observe adhesion between the cecum and peritoneum. Severe adhesion was observed in the control group, with the cecal tissue surface tightly adhered to the peritoneum. In contrast, the PTL@GC group showed significantly reduced tissue adhesion, likely due to its excellent tissue adhesion properties, forming a stable bond with the injured tissue (Figure 7A,B and Figure S15). The therapeutic effect of PTL@GC film on postoperative adhesion between the cecum and abdominal wall was characterized using hematoxylin-eosin (H&E) staining, immunofluorescence (IF) staining, and Masson’s trichrome staining. The results of H&E staining (Figure 7C,D) showed that there was severe adhesion between the cecum and the abdominal wall in the control group. The adhesion area accounted for approximately 24.22% of the total abdominal cavity area, and a layer of fibrous tissue was formed between the damaged cecum and the inner muscle layer of the abdominal wall. In contrast, in the mice treated with the PTL@GC film, the adhesion area accounted for approximately 1.44% of the total abdominal cavity area, and no adhesion or blood vessels were observed. The adhesion area was reduced by approximately 22.77% compared with that of the control group. Notably, the PTL@GC film exhibited excellent tissue adhesion, remaining attached to the cecal surface after 7 days of treatment. Additionally, collagen deposition was observed in the control group, forming a dense connective tissue layer and leading to adhesion between the cecum and peritoneum, contributing to postoperative tissue adhesion. Masson’s trichrome staining (Figure 7E) showed a significant increase in collagen in the adhesion area, and immunohistochemical staining revealed increased α-smooth muscle actin (α-SMA) around blood vessels (Figure 7F), indicating enhanced cellular activity and fibrotic response. Conversely, mice treated with PTL@GC film exhibited significantly reduced collagen fibers and blood vessels, attributed to its effective anti-adhesion properties.

To further explore the anti-inflammatory properties of PTL@GC film in vivo, immunofluorescence (IF) staining was performed for the pro-inflammatory markers CD68^+^ and the inflammatory cytokine TNF-α in macrophages. The control group showed a higher number of CD68^+^ macrophages, indicating an inflammatory response. In contrast, tissues treated with PTL@GC film exhibited significantly lower expression of CD68^+^ and TNF-α compared to the control group, attributed to the anti-inflammatory activity of CAT (Figure 7G). These results demonstrate that PTL@GC effectively reduces adhesion between the damaged cecum and peritoneum.

### 3.6. PTL@GC Alleviates Hypoxia and Induces Disulfidptosis to Inhibit Postoperative Colorectal Cancer

**Figure 8:**
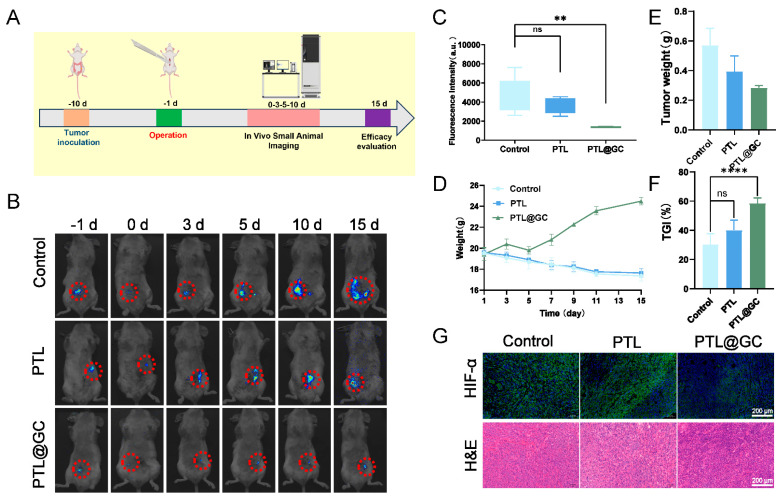
Antitumor therapeutic efficacy of PTL@GC. (**A**) Timeline of the animal experiment. (**B**) In vivo tumor imaging of mice in different groups on days −1, 0, 3, 5, 10, and 15. The red circle indicates the tumor signal. (**C**) Statistical analysis of tumor fluorescence in different groups after treatment. (**D**) Statistical analysis of body weight changes in mice during the treatment process. (**E**) Statistical analysis of tumor weight in different groups after treatment. (**F**) Statistical analysis of tumor inhibition rate in different groups after treatment. (**G**) HIF-1α, H&E, and TUNEL staining of tumors in different groups after treatment. ns: *p* > 0.05, ** *p* < 0.01, **** *p* < 0.0001.

To evaluate the anticancer efficacy of PTL@GC film in clinical practice, an orthotopic colon cancer model was established by injecting SW48-luc cells into the colorectal wall of BALB/c mice. On day 10 after tumor inoculation, the successfully modeled mice were randomly divided into three groups. The tumors were surgically removed, and the film was adhered between the surgical bed and peritoneum after hemostasis (Figure 8A).

Tumor burden was monitored using an in vivo imaging system, and bioluminescence intensity was quantified using IVIS Version 3.0 software (Seoul, Republic of Korea) to assess tumor load (Figure 8B). Fifteen days after film implantation, five mice from each group were euthanized, and residual tumors were collected and weighed (Figure 8C). The results showed that PTL@GC film had a more effective inhibitory effect compared to the control group (tumor burden: approximately 28.42-fold vs. 56.96-fold). The body weight of mice in the PTL@GC group increased steadily and slowly throughout the treatment, while the control and PTL groups showed weight loss (Figure 8D). Among all groups, PTL@GC exhibited the strongest tumor growth inhibition, with a tumor inhibition rate of 58.49% (Figure 6F and Figure 8E). Histological analysis (H&E, TUNEL, and HIF-1α staining) (Figure 8G and Figure S16) revealed increased TUNEL expression and decreased HIF-1α expression in the PTL@GC group, indicating that the film inhibited tumor growth and alleviated the hypoxic microenvironment. Overall, these results confirm that PTL@GC inhibits tumor growth by alleviating hypoxia and inducing disulfidptosis.

## 4. Discussion

The high recurrence rate and high incidence of abdominal adhesions following CRC surgery present significant clinical challenges. Traditional anti-adhesion materials, such as polyethylene glycol hydrogels and chitosan films, are restricted by insufficient mechanical properties, low drug release efficiency, and short in vivo retention times. As a result, it is difficult for them to meet the dual requirements of anti-adhesion and anti-recurrence. Moreover, existing tumor treatment materials often lack tissue specificity or cannot adequately adapt to the tumor microenvironment, leading to limited therapeutic effects. Janus materials, with their dual-sided heterogeneous structure, can optimize physical barriers and bioactive interfaces, enabling synergistic anti-adhesion and anti-recurrence effects [38]. Our research group has developed a novel Janus protein nanofilm (PTL@GC) based on amyloid-like protein film self-assembly technology. This material is formed by one-step co-loading of GOx and CAT using a self-assembly system of lysozyme solution and cysteine, resulting in a stable hydrophobic-hydrophilic heterogeneous structure [39]. The PTL@GC film exhibits excellent mechanical stability, drug delivery efficiency, and biocompatibility, providing an innovative solution for post-CRC surgery treatment.

Leveraging the unique advantages of Janus materials, our research group has developed the PTL@GC Janus protein nanofilm, which achieves functional synergy between anti-adhesion and anti-recurrence through hydrophobic-hydrophilic partitioning. The hydrophobic side, rich in hydrophobic residues such as Val and Leu, adheres tightly to the intestinal tissue surface via hydrophobic interactions. This ensures stable attachment in the dynamic intestinal environment and prevents displacement caused by intestinal peristalsis or fluid scouring. This strong adhesion enables sustained, targeted release of GOx and CAT to the tumor resection site, avoiding drug diffusion to non-target areas and enhancing the precise killing of residual tumor cells [40]. Experiments show that the PTL@GC film maintains over 80% of its adhesion area after 15 days in an in vitro intestinal motility simulation, with a friction coefficient of 0.165 and only a 5.4% decrease in contact angle, supporting long-term drug release (60% release over 4 weeks). Stable adhesion of the hydrophobic side is a key prerequisite for GOx-induced disulfidptosis. By continuously delivering GOx to the tumor microenvironment, NADPH is depleted, inducing disulfidptosis in SLC7A11-high tumor cells. Meanwhile, the hydrophilic region, rich in Glu and Lys residues, inhibits fibrinogen adsorption and fibroblast migration through electrostatic repulsion, reducing postoperative fibrosis. CAT on the hydrophilic side clears H_2_O_2_, alleviating oxidative stress and further inhibiting inflammation-driven adhesion formation. In a cecum abrasion model, the PTL@GC group showed a 22.77% reduction in adhesion area, with significantly decreased collagen deposition and α-SMA expression, indicating that the hydrophilic side inhibits adhesion through both physical isolation and bioactive regulation, such as CAT-mediated ROS clearance. Furthermore, the Janus structure achieves functional synergy between GOX and CAT through spatial partitioning: the hydrophobic side targets GOx delivery to induce tumor disulfidptosis, while the hydrophilic side clears H_2_O_2_ and alleviates oxidative damage, avoiding the side effects caused by H_2_O_2_ accumulation in traditional single-function materials, such as GOx-only films. This in situ generation and clearance strategy not only enhances anti-tumor efficacy but also significantly suppresses postoperative adhesion. The PTL@GC film also outperforms traditional Janus materials in structural stability and biocompatibility. Traditional materials rely on chemical modifications, such as plasma treatment, to achieve functional partitioning, which often leads to functional decay due to weak interfacial bonding [41]. For example, CA/PVDF film experiences a flux decline of over 50% after 2 h of operation [42]. In contrast, PTL@GC film forms a stable heterogeneous structure through the self-assembly of lysozyme β-sheet hydrogen-bond networks, with a uniform thickness of 620 nm, requiring no exogenous cross-linkers. Its interfacial bonding energy is significantly enhanced, with adhesion strength three times higher than that of dopamine coatings [43]. Additionally, the natural lysozyme matrix degradation products are non-cytotoxic, with a hemolysis rate of less than 1% and a degradation cycle of 50 days, synchronized with tissue repair, avoiding the toxicity risks associated with traditional metal-polymer composites.

Experimental data further confirm the applicability of PTL@GC in preventing adhesion and recurrence [44]. In terms of the anti-adhesion efficacy, the PTL@GC group demonstrated a significant reduction in the number of CD68^+^ macrophages and the expression of TNF-α, indicating that it effectively inhibits inflammation in the microenvironment and thus reduces adhesion through CAT-mediated ROS scavenging combined with the physical barrier on the hydrophilic side [45]. The PLCL/GelMA composite film developed by the Gao team reduces collagen deposition by activating the fibrinolytic system and promoting the secretion of MMP-9. In contrast, the PTL@GC film developed in this study was designed from the perspective of anti-oxidation, inhibiting the release of pro-inflammatory factors such as TNF-α by scavenging ROS. Together, these two studies reveal the crucial role of “inflammatory regulation–ECM metabolism” in adhesion formation. It is worth noting that the design concept of the PLCL film in Gao’s study, which provides mechanical support, and GelMA, which endows bioactivity, is similar to the dual-functional mechanism of “physical isolation–dynamic anti-oxidation” of PTL@GC, both reflecting the concept of “combining barrier function with biological intervention”. In the cecum abrasion model, compared with the control group, the adhesion area of the PTL@GC group decreased by 22.57%, and the deposition of collagen and the expression of α-smooth muscle actin (α-SMA) also significantly decreased, outperforming the materials based on hyaluronic acid. In the cecum abrasion model, the PTL@GC group exhibited a 22.77% reduction in adhesion area compared to the control group, with significantly decreased collagen deposition and α-SMA expression, outperforming hyaluronic acid-based materials. In terms of anti-recurrence efficacy, in an orthotopic tumor model, PTL@GC reduced the tumor burden to 28.42% of the control group, with enhanced TUNEL apoptosis signals and decreased HIF-1α expression, indicating that the Janus structure effectively alleviates hypoxia and induces tumor cell death through the GOx-CAT cascade [46].

In summary, the PTL@GC Janus protein nanofilm, through its structural design, enzyme metabolic regulation, and biodegradable properties, provides an innovative solution for dual complications following CRC surgery. Its excellent performance in preclinical models, with a hemolysis rate of <1%, a degradation cycle synchronized with tissue repair (complete metabolism in 50 days), and no pathological damage observed in vital organs, demonstrates good biosafety. Future research could further explore modular functional designs, such as integrating immune modulators and personalized customization, to adapt to the metabolic characteristics of different tumor subtypes, thereby expanding its potential in precision medicine. Additionally, its efficacy should be validated in large animal models, and large-scale production processes and intraoperative fitting precision should be optimized to ensure clinical applicability.

## 5. Conclusions

This study developed an innovative Janus protein nanofilm (PTL@GC) that effectively alleviates hypoxia in the tumor microenvironment and inhibits recurrence through enzyme cascade reactions and disulfidptosis mechanisms, while significantly reducing postoperative abdominal adhesions. The PTL@GC film forms a stable Janus structure through protein self-assembly, requiring no complex exogenous modifications, and exhibits high biocompatibility and controllable degradability. Its hydrophobic side firmly adheres to the intestinal surface, preventing displacement, while the hydrophilic side isolates abdominal organs through a hydration layer, reducing fibrin deposition and significantly lowering the adhesion risk. The synergistic effects of GOx and CAT in PTL@GC induce tumor cell disulfidptosis and alleviate hypoxia, while reducing oxidative stress and inflammation. Animal experiments demonstrate the significant efficacy of PTL@GC in anti-adhesion and anti-tumor applications, along with its excellent biocompatibility and degradability. This material provides an innovative solution for dual complications following CRC surgery, and further optimization of its design could advance its application in precision medicine.

## Data Availability

The data that support the findings of this study are available from the corresponding author upon reasonable request.

## Supplementary Materials

The following supporting information can be downloaded at: https://www.mdpi.com/article/10.3390/nano15090670/s1.

## Abbreviations

The following abbreviations are used in this manuscript:

CRC	Colorectal Cancer
GOx	Glucose Oxidase
CAT	Catalase
ROS	Reactive Oxygen Species
NADPH	Nicotinamide Adenine Dinucleotide Phosphate
IL-6	Interleukin-6
SEM	Scanning Electron Microscopy
XPS	X-ray Photoelectron Spectroscopy
CD	Circular Dichroism
MALDI-TOF	Matrix-Assisted Laser Desorption/Ionization Time-of-Flight
