# Supplementary material for: A Janus Amyloid-like Nanofilm Inhibits Colorectal Cancer Postoperative Recurrence and Abdominal Adhesion via Synergistic Enzyme Cascade
Source: Nanomaterials (Basel). 2025 Apr 28;15(9):670. doi: 10.3390/nano15090670 (PMC12073602; doi:10.3390/nano15090670)
Supplement: Supplementary file 1 [file nanomaterials-15-00670-s001.zip › nanomaterials-3620773-supplementary.pdf]

Supporting information

Man Zhang [1], Junhao Kou [2], Zhengyi Song [2], Ling Qiu [1], Chunzhao Yang [1] and Qi Xue [1,*]

1 Southern Medical University Hospital of Integrated Traditional Chinese and Western Medicine, Southern Medical University, Department of General Surgery, Guangdong 510315, China

2 College of Pharmacy, Xi'an Medical University, Xi'an 710021, China

* Correspondence: xueqi@smu.edu.cn

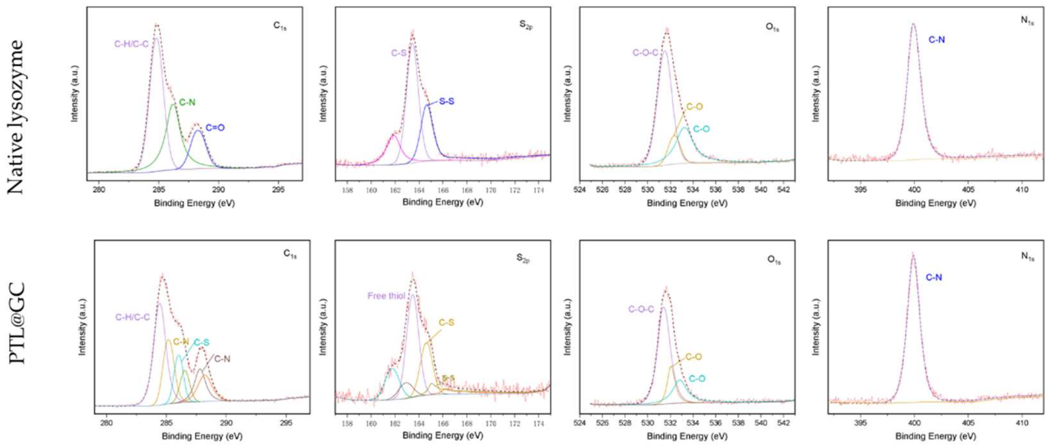

Figure S1. XPS analysis of the chemical bonding states of native lysozyme and PTL@GC. High-resolution spectra of C1s, S2p, O1s, and N1s.

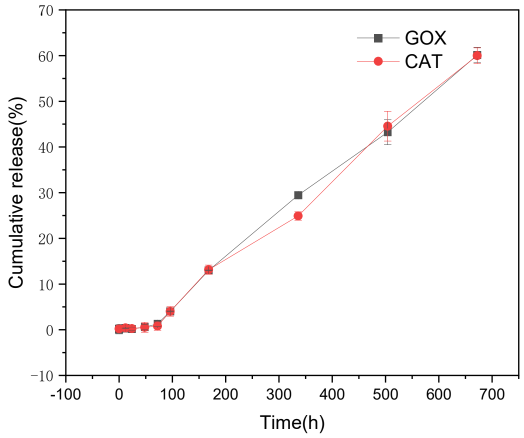

Figure S2. In vitro drug release profile of GOx-CAT. The cumulative percentage (%) of drug release within 700 hours. The data are presented as mean ± standard deviation (n = 3). Sustained release kinetics were observed, with more than 20% of the drug released within 300 hours.

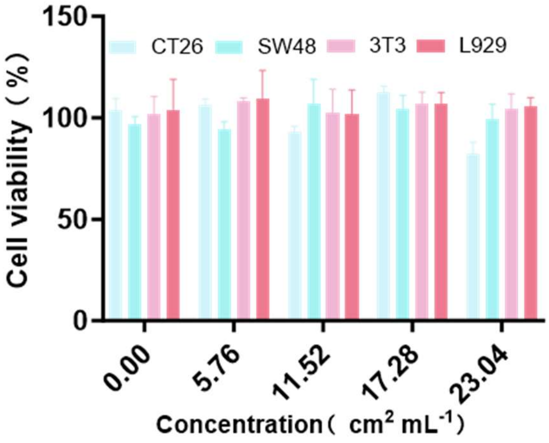

Figure S3. Cytotoxicity evaluation of PTL. Cell viability (%) of the treatment groups with different concentrations (0 – 23.04 cm^2 mL^{-1}). PTL shows good biological safety.

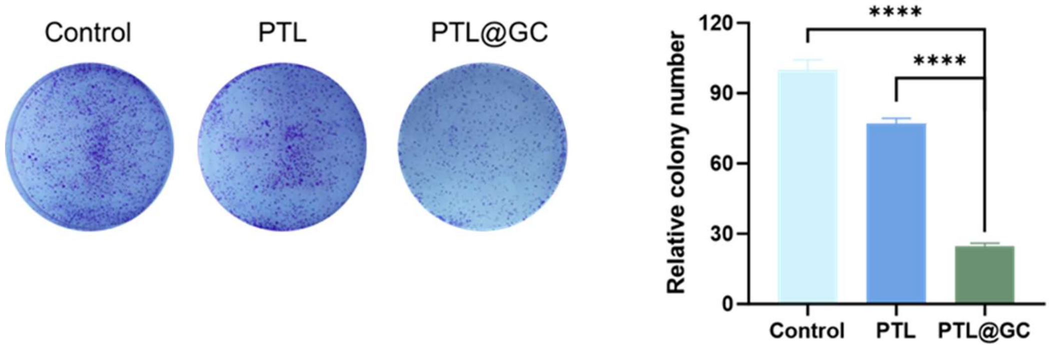

Figure S4. Colony formation assay. Representative images of colony formation in the Control group, PTL group, and PTL@GC group. PTL@GC shows a significantly reduced colony formation ability (n = 3, $^{*}p$<0.05, $^{**}p$<0.01, $^{***}p$<0.001, $^{****}p$<0.0001, $^{*****}p$<0.00001).

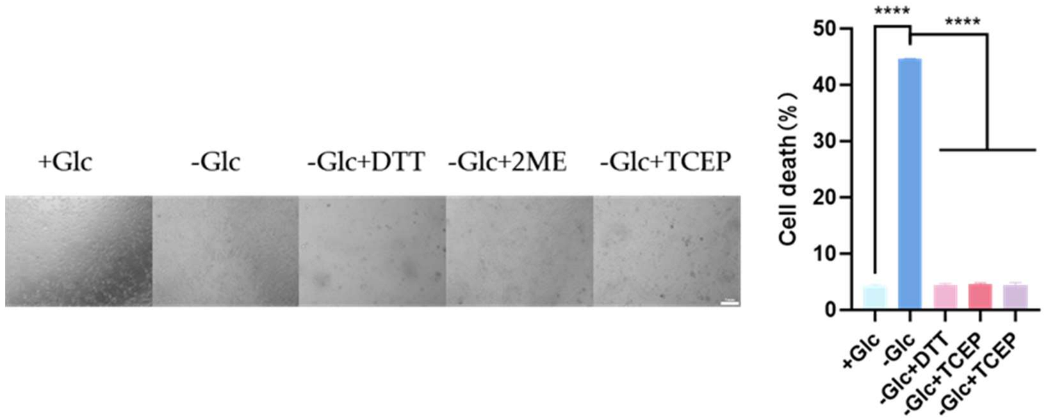

Figure S5. Percentage of cell death in each group: +Glc (Control group), -Glc (starvation group), -Glc plus DTT, 2ME, or TCEP (reducing agents). The data show that reducing agents can alleviate cell death (n = 5, $^{*}p$<0.05, $^{**}p$<0.01, $^{***}p$<0.001, $^{****}p$<0.0001, $^{*****}p$<0.00001).

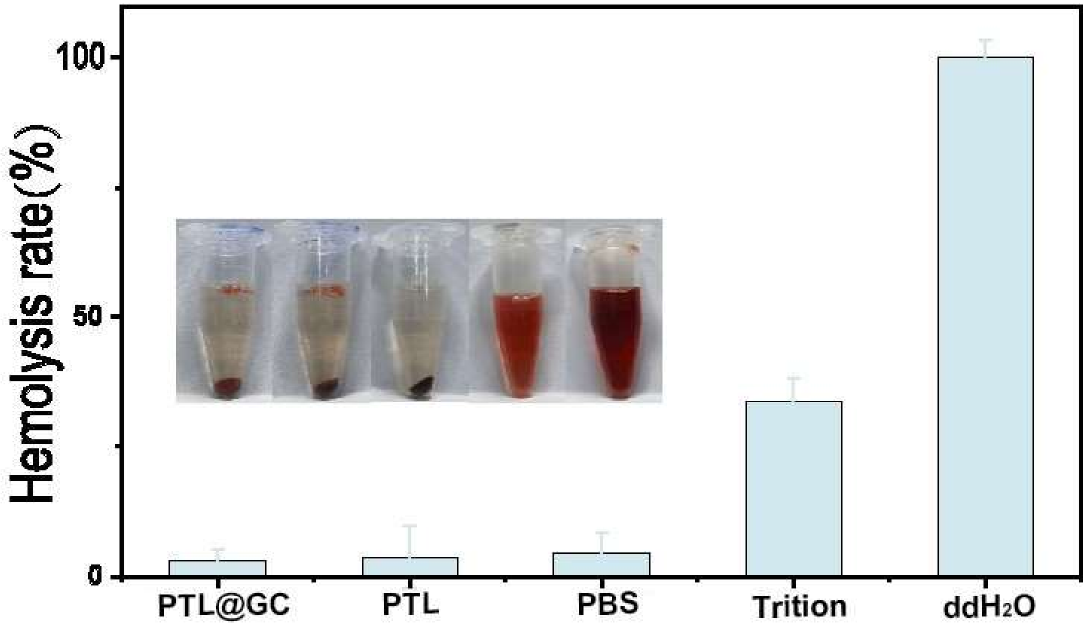

Figure S6. Hemolysis experiment. The samples are, respectively, PTL@GC, PTL, PBS (negative control), Triton X-100, and H_2O (positive control). The hemolysis rates of both PTL@GC and PTL are within the normal range.

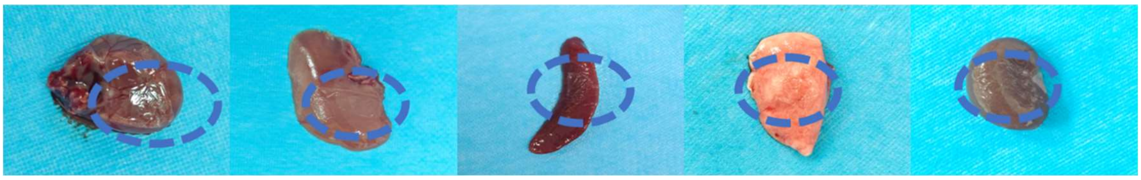

Figure S7. Adhesion experiment of PTL@GC. PTL@GC can stably adhere to the heart, liver, spleen, lung, and kidney.

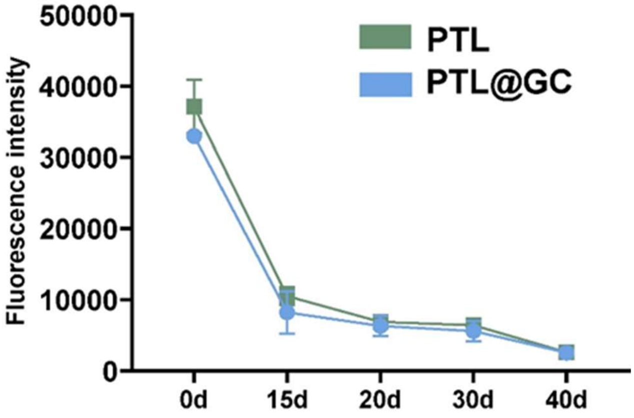

Figure S8. In vivo fluorescence tracing and degradation analysis of IR783-labeled PTL and PTL@GC. Fluorescence decay of PTL and PTL@GC over time.

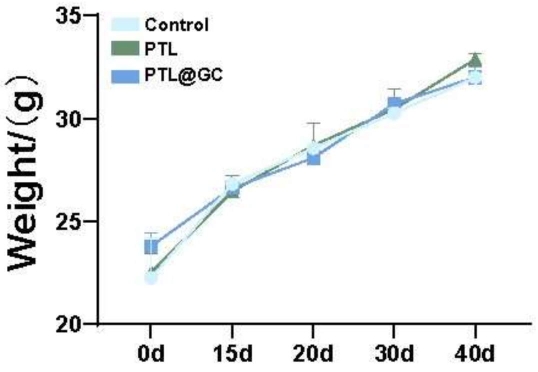

Figure S9. The body weights of the mice in the Control group, the group with in situ implanted PTL, and the group with in situ implanted PTL@GC increased steadily.

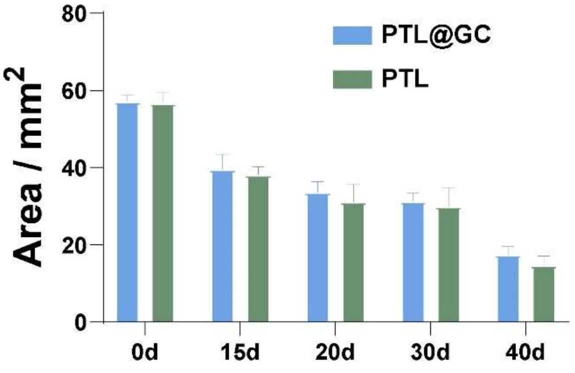

Figure S10. Quantification diagram of the degradation area of IR783-labeled PTL and PTL@GC over time.

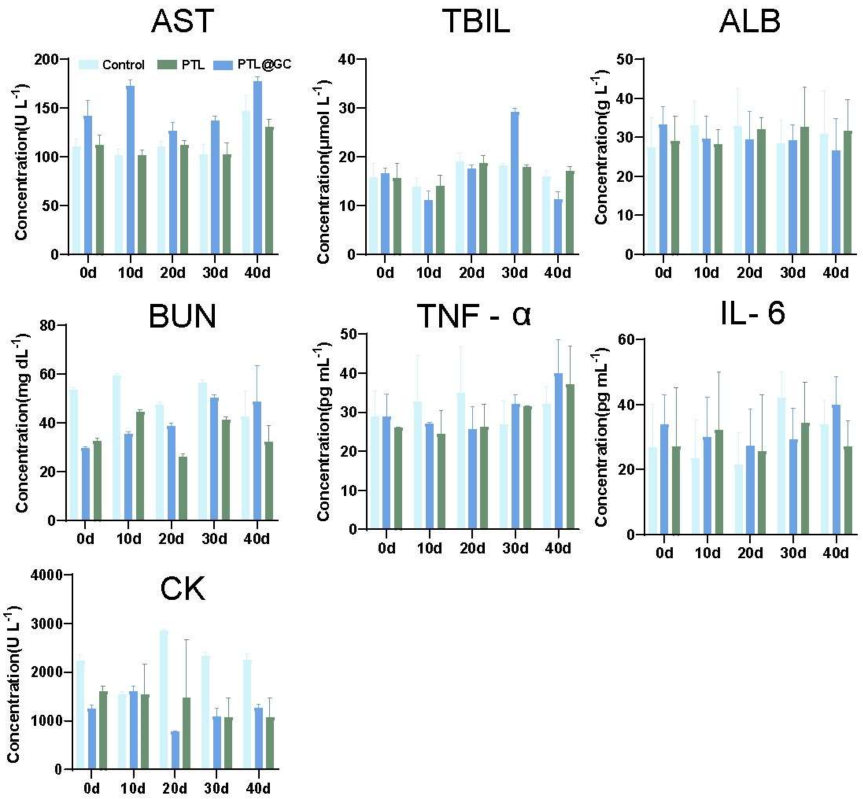

Figure S11. The levels of AST, TBIL, ALB, BUN, TNF-α, IL-6, and CK in mice from the PTL group and the PTL@GC group within 40 days, compared with those of the Control group, and the reference values of medical standards. The levels in all three groups are within the normal range.

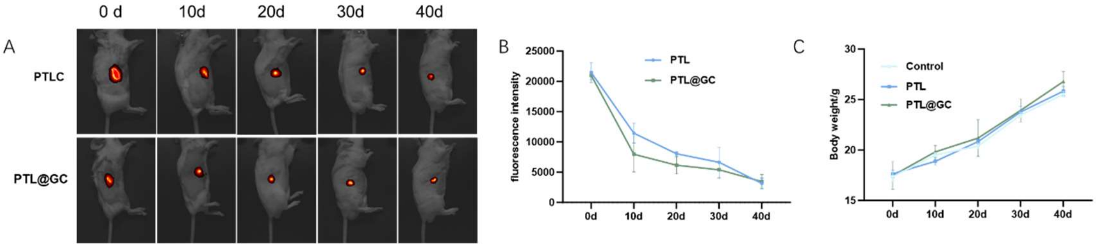

Figure S12. Subcutaneous in vivo fluorescence tracing and degradation analysis.

A) The fluorescence of PTLC and PTL@GC decays slowly over time.

B) Changes in fluorescence intensity.

C) Changes in the body weight of mice.

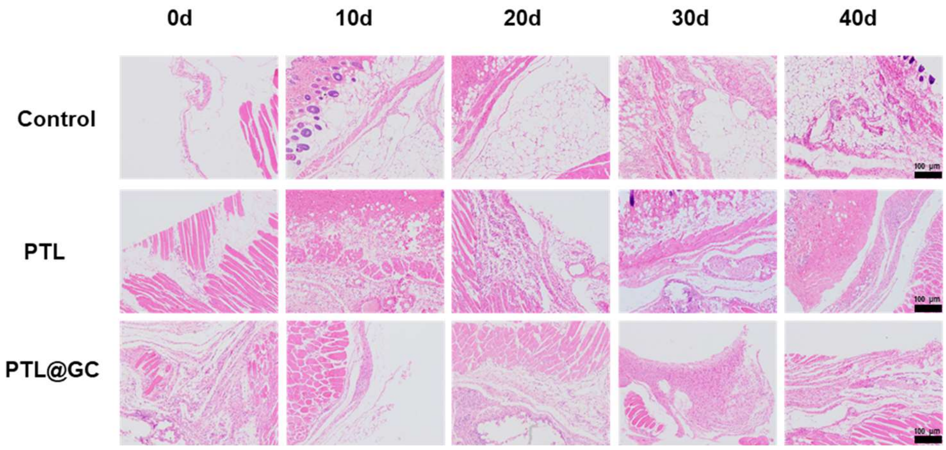

Figure S13. Results of subcutaneous H&E staining after implantation in the Control group, the PTL group, and the PTL@GC group. H&E-stained sections from the Control group, the PTL group, and the PTL@GC group. There is no tissue damage in any group.

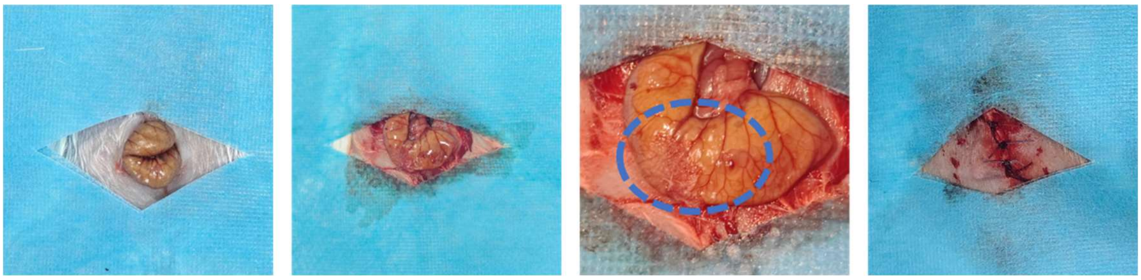

Figure S14. Schematic diagram of the construction of the cecal injury model and application of the membrane.

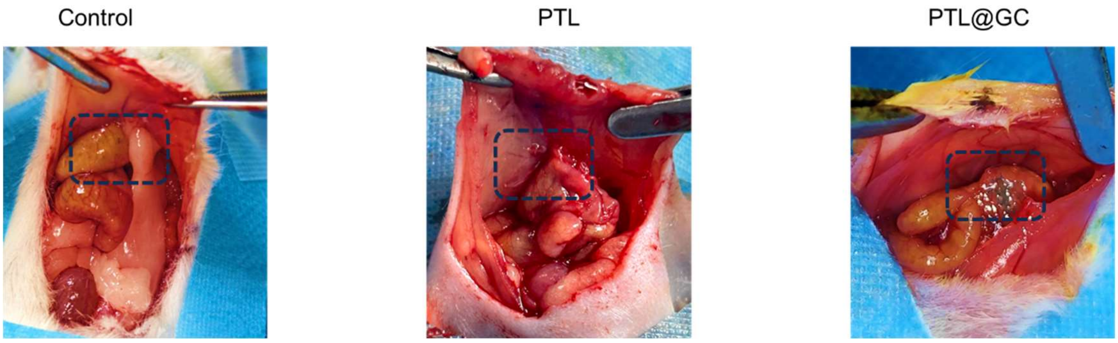

Figure S15. Antiadhesion effects of the Control group, the PTL group, and the PTL@GC group. The PTL@GC group shows an adhesion inhibition effect.

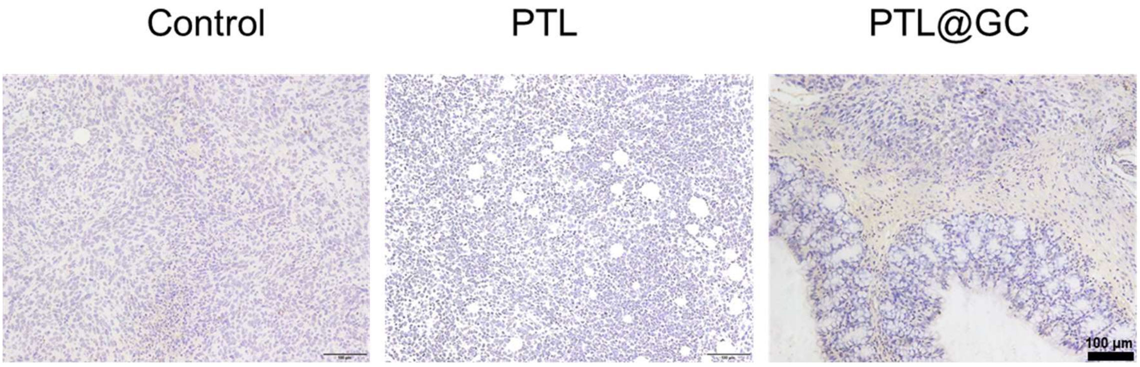

Figure S16. Results of tumor H&E staining. H&E-stained sections of tumor tissues in the Control group, the PTL group, and the PTL@GC group.
